# Barriers to Gambling Treatment Among American Military Personnel: A Qualitative Study

**DOI:** 10.1007/s10899-025-10385-z

**Published:** 2025-04-24

**Authors:** Noa Vana, Shane W. Kraus, Bailey M. Way, Todd L. Jennings, Belle Gavriel-Fried

**Affiliations:** 1 The Bob Shapell School of Social Work, Tel Aviv University, Tel Aviv, Israel; 2 Department of Psychology, University of Nevada, Las Vegas, Las Vegas, NV USA

**Keywords:** Barriers to treatment, Active duty, Military veterans, Gambling disorder

## Abstract

Gambling disorder (GD) poses a significant public health problem, with treatment access frequently hindered by barriers. This study sought to identify the unique internal and external barriers encountered by military personnel with GD using a qualitative descriptive method. Twenty-eight United States military veterans and service members (SMs) were recruited through purposeful sampling strategies and interviewed using a semi-structured interview format. Content analysis revealed two main themes: “*Emotional Suppression in Military Culture*” reflecting military cultural norms that emphasize strength, discipline, and emotional control, which were internalized by the participants and created significant internal barriers; “*Structural Ignorance of Gambling Problems*” uncovers external barriers such as the normalization of gambling, insufficient recognition of gambling’s addictive nature within military and Veterans Affairs (VA) settings, and inadequate treatment options within the VA healthcare system. The study underscores the complex interplay between cultural norms and institutional practices and proposes policy recommendations to improve help-seeking behaviors for veterans and SMs.

## Introduction

Gambling disorder (GD) is a growing public health concern worldwide, creating significant negative consequences for individuals, families, and the society at large (Abbott, 2020). It refers to a pattern of repeated betting and wagering that continues despite creating multiple problems in an individual’s life (American Psychiatric Association, 2022). The estimated lifetime prevalence of GD in the USA ranges from 0.42 to 4.0% (Black & Shaw, 2019). Notably, GD can be effectively treated with pharmacological and psychosocial interventions (Etuk et al., 2020; Pickering et al., 2020); however, accessing treatment is often hindered by internal barriers, such as denial, low motivation, fear of stigma, and shame (Barnett et al., 2021; Cernasev et al., 2021), as well as external barriers, like inadequate insurance, financial constraints, and limited access to trained professionals (Ocloo et al., 2021; Sheikh et al., 2021; Troup et al., 2021).

Recent literature focuses on the internal and external barriers that hinder access to treatment for GD (Quigley, 2022; Ribeiro et al., 2021; Stenbro et al., 2023), emphasizing the need to address these challenges in different socio-cultural contexts (Håkansson et al., 2024). Studies underscore the vulnerability of veterans and SMs to developing GD compared to the general population (Etuk et al., 2020; Levy & Tracy, 2018), in part due to veterans’ and SMs’ unique characteristics, such as suffering from higher rates of posttraumatic stress disorder (PTSD), depression, and substance use disorders (SUDs) related to their military service (Grubbs et al., 2023; Shepherd-Banigan et al., 2023; Silvestrini & Chen, 2023). Nonetheless, only a handful of studies have explored the barriers to GD treatment among veterans, with GD often being overlooked in clinical screenings (Kraus et al., 2020). Thus, our study sought to identify the unique internal and external barriers encountered by U.S. military personnel living with GD.

### Internal and External Barriers to Help-Seeking for Gambling Disorder

Existing literature suggests substantial internal and external barriers to accessing healthcare for various health conditions, including GD (Daugelat et al., 2023; Håkansson et al., 2024). Internal barriers refer to personal attitudes and psychological obstacles individuals face when considering seeking treatment for their health problems (Sarkar et al., 2021), including denial of an existing problem, insufficient motivation for treatment (Barnett et al., 2021), feelings of shame, and fear of social stigma (Cernasev et al., 2021). These feelings might be triggered by various external factors, such as cultural and social contexts (Schuler et al., 2015).

A growing body of literature is beginning to connect internal and external barriers to accessing treatment specifically for GD. A notable internal barrier is that individuals with GD often prefer to manage their gambling issues on their own and struggle with feelings such as denial, shame, secrecy, and fear of stigma (Quigley, 2022; Stenbro et al., 2023). The psychological impact of GD, combined with stress from debts and strained relationships, can lead to a state of denial or a belief that one can “gamble their way out” of the situation, entrenching the gambling behavior and creating a reluctance to seek help (Ribeiro et al., 2021). Feelings of hopelessness predict an escalation in gambling problems (Otis et al., 2021), and in combination with other emotions, such as shame, can further delay seeking treatment (Estévez et al., 2023, 2024). External barriers to seeking treatment for GD involve difficulties with the organization and availability of treatment, as well as societal and regulatory factors that hinder access to care. For example, access to treatment can be limited by geographical factors. Specialized services for GD may not be available in all regions, making it difficult for individuals in remote or underserved areas to access care (Håkansson et al., 2024).

Individuals with GD may experience a variety of internal and external barriers to accessing treatment. However, there has been little attention surrounding the unique experiences of veterans and SMs with accessing treatment for GD. Given that military personnel have a unique socio-cultural context relative to civilians and endorse higher rates of GD at the population-level, it is pertinent to consider the unique internal and external barriers this population may encounter when seeking services for GD (Stefanovics et al., 2023).

### The Socio-Cultural Context of US Military Veterans and Service Members

Studies examining military culture observe that veterans and SMs often hold values that impede help-seeking, which presents notable internal barriers to addressing mental health concerns (Randles & Finnegan, 2022; Silvestrini & Chen, 2023), including addictions (Edwards et al., 2021; Kiernan et al., 2018). For example, military culture promotes stoicism and self-reliance, prioritizing mission accomplishment over personal discomfort and labeling sickness as a sign of weakness, which ultimately discourages veterans and SMs from seeking help (Randles & Finnegan, 2022). Veterans also face unique health-related challenges, such as higher rates of morbidity, physical impairment, PTSD, depression (Shepherd-Banigan et al., 2023; Silvestrini & Chen, 2023), and SUDs (Grubbs et al., 2024). The transition to civilian life for veterans often involves several external barriers that can hinder their ability to seek help. One major challenge is often the struggle with the loss of their former structured environment and camaraderie they experienced in the military, leading to feelings of isolation and identity confusion. Navigating civilian life requires them to translate their military skills to civilian job markets, leading to underemployment and economic difficulties (Adams et al., 2021). They may also encounter logistical barriers, such as long appointment waiting times, difficulty accessing Veterans Affairs (VA) services, or being unaware of available services altogether (Silvestrini & Chen, 2023).

Despite evidence that SMs and veterans are at increased risk of experiencing GD (Etuk et al., 2020; Metcalf et al., 2022) and co-morbid psychiatric disorders (Fogle et al., 2020), few studies have addressed the barriers these populations may experience in accessing GD treatment (e.g., Kraus et al., 2020; Shirk et al., 2022). To our knowledge, no studies have qualitatively explored barriers to GD treatment in U.S. veterans and SMs, though prior work has examined challenges faced by veterans reporting issues with addictions (Champion et al., 2022). The present study poses one main research question: What are the unique internal and external barriers to GD treatment that veterans and SMs in the U.S. experience? Through this work, we seek to gain insights needed to promote practical recommendations for clinicians and policymakers and enhance the help-seeking behaviors of veterans and SMs.

## Method

### The Methodological Approach

A descriptive qualitative method was utilized (Sandelowski, 2010). This method, widely applied in healthcare settings, is also employed regularly in mental health research because it offers rich and direct accounts of individuals’ perceptions and experiences of a given phenomenon or event. Specifically, it is characterized by minimal interpretation, and therefore, it remains closely aligned with the data throughout the analytical process (Colorafi & Evans, 2016). Due to the straightforward nature of this method, it is particularly suitable for capturing precise accounts of perceptions and experiences of phenomena, resulting in findings that are comprehensible to vulnerable populations and clinical practitioners (Sullivan-Bolyai et al., 2005). Hence, we believe the descriptive qualitative method was most suitable for the current study.

### Procedure and Participants

Semi-structured interviews were conducted with 28 U.S. military veterans and SMs. A combination of two purposeful sampling strategies (Patton, 2015) were employed: criterion-based case selection and maximum variation sampling. The eligibility criteria required participants to be either currently serving in the U.S. Armed Forces or have previously served but had been formally discharged (veterans) from the military, be at least 18 years of age, and endorse at least four criteria of the DSM-5 GD diagnosis (American Psychiatric Association, 2013).

Recruitment efforts to ensure a comprehensive and representative sample of military veterans and SMs involved distributing flyers in community settings, using social media platforms, and engaging influential figures within the veteran community. Additionally, clinicians, medical centers, and organizations supporting veterans were contacted to distribute information on the study. Participants were recruited nationwide until data saturation was achieved.

Upon obtaining informed consent, the interviews occurred over confidential Zoom meetings, which were recorded and transcribed verbatim. Using a semi-structured interview schedule to elicit rich data (Bearman, 2019), participants were asked broad questions to explore their experiences relating to barriers to treatment for GD (e.g., “Please describe the barriers you encounter while seeking treatment for your gambling problem”). Participants were compensated for their time with $75 gift cards. The interviews were conducted from August 2023 to August 2024. The study was approved by the Institutional Review Board of XX. Overall, as can be seen in Table 1, a diverse sample of 28 participants was obtained, including 24 veterans and 4 SMs, with an average age of 46.5 years (ranging from 27 to 73). All names used in the paper are pseudonyms.

**Table 1:** Illustration of the Research Sample

Name	Age	Veteran /SM	Gender	Marital Status	Recovered/ Non-Recovered	Race	Sexual Orientation	Military Branch
Brayden	57	Veteran	Male	Single	Non-Recovered	White	Heterosexual	Navy
Britney	37	SM	Female	Divorced	Non-Recovered	Black	Bisexual	Navy
Claire	56	Veteran	Female	Single	Non-Recovered	Black	Heterosexual	Air Force
Cody	29	Veteran	Male	Relationship	Non-Recovered	Black	Heterosexual	Marines
Cristina	54	Veteran	Female	Single	Non-Recovered	Black	Heterosexual	Navy
David	45	Veteran	Male	Divorced	Recovered	White	Heterosexual	Air Force Reserves
Doug	59	Veteran	Male	Married	Non-Recovered	White	Heterosexual	Air Force
Drake	72	Veteran	Male	Divorced	Non-Recovered	White	Heterosexual	Air Force
Edward	51	Veteran	Male	Divorced	Non-Recovered	Asian	Heterosexual	Army
Elliot	28	SM	Male	Married	Non-Recovered	Black	Heterosexual	Army
Freya	31	SM	Female	In a relationship (Divorced previously)	Non-Recovered	White	Heterosexual	Army
Grace	53	Veteran	Female	Divorced	Non-Recovered	Mixed	Nonsexual	Army
Jack	38	Veteran	Male	Divorced	Non-Recovered	Asian	Heterosexual	Navy
Jared	33	Veteran	Male	Single	Non-Recovered	Mixed	Heterosexual	Marines
Johnny	48	Veteran	Male	Divorced	Non-Recovered	White	Heterosexual	Marines
Kaleb	37	Veteran	Male	Married	Recovered	White	Heterosexual	Air Force
Lucas	41	Veteran	Male	Married	Recovered	White	Heterosexual	Air Force
Madison	42	Veteran	Female	Relationship	Non-Recovered	White	Lesbian	Air Force
Maria	50	Veteran	Female	Divorced	Recovered	Mixed	Heterosexual	Army
Mark	51	Veteran	Male	Divorced	Non-Recovered	White	Heterosexual	Army
Mason^a^	55	Veteran	Male	?	Non-Recovered	Latino	Heterosexual	Navy
Nate	59	Veteran	Male	Divorced	Non-Recovered	White	Heterosexual	Army
Peter	64	Veteran	Male	Divorced	Non-Recovered	White	Heterosexual	Army
Robert	35	Veteran	Male	Married (Divorced previously)	Recovered	White	Heterosexual	Army
Ryan	73	Veteran	Male	Single	Non-Recovered	Mixed	Gay	Air Force
Sherry	39	Veteran	Female	Single	Non-Recovered	Black	Heterosexual	Air Force
Wyatt	38	Veteran	Male	Divorced	Non-Recovered	Native American	Heterosexual	Marines
Zach	27	SM	Male	Married	Recovered	White	Heterosexual	Air Force

^a^There is missing data for one participant who did not complete the follow-up after their participation in the main interview. SM = Service Member

### Data Analysis

Content analysis is recommended for data analysis when using the descriptive qualitative method (Sandelowski, 2010). In the current study, we used directed (deductive) content analysis—using a pre-existing coding system based on the literature representing various internal and external barriers to treatment (e.g., shame, accessibility), and conventional (inductive) content analysis based on codes and categories that emerge from the data (Hsieh & Shannon, 2005; Patton, 2014). Directed content analysis is applied when there is an established theory that could be further elaborated in a different context, while conventional or inductive content analysis is utilized when there is limited existing research (Elo & Kyngäs, 2008; Hsieh & Shannon, 2005). For the current study, we found both approaches suitable. Given the existing knowledge surrounding barriers to treatment for addictions, directed content analysis was appropriate for coding the initial interviews. However, less is known about the unique barriers encountered by U.S. military veterans and SMs seeking treatment for GD, which necessitated the use of conventional content analysis as new codes and categories emerged from the data. According to Sandelowski (2010), who initiated the descriptive qualitative approach, when researchers decide to start the analysis by implementing pre-existing coding systems, such coding systems are often modified or even completely replaced during the analysis process because new codes have emerged from the data.

Specifically, after reading the interviews, we chose five interviews of participants from diverse backgrounds and military experiences and analyzed them using the coding system. At the same time, while reading the interviews, we also labeled new codes reflecting unique aspects of veterans’ and SMs’ experiences that were not included in our initial coding system. After five interviews, we amended the initial coding system, and the additional 23 interviews were analyzed accordingly. Again, segments that did not appear in the amended coding system were then labeled. As can be seen, the analysis began by using directed content analysis and later transitioned to conventional content analysis (Armat et al., 2018). At the end of this process, 57 codes were generated. All data within each code were reviewed, discussed, and refined by the research team. Codes were merged and conceptualized into nine categories according to differences and similarities in the content and then consolidated into five categories (Hsieh & Shannon, 2005). In the last stage, these categories were formulated and grouped into two final main themes to encapsulate the comprehensive meaning derived from the codes. Detailed information on themes, categories, and codes is provided in Table 2.

**Table 2:** Illustration of the Barriers to Treatment among Veterans and SMs: Theme, Categories, and Codes

Themes	Categories	Codes
Emotional Suppression in the military culture	“You Live Like a Strong and Not Weak”	Military culture does not allow weakness, Socialization to being strong, Self-stigma, Shame, Public stigma, Double stigma
	“You Can Get It Flagged”	You can get it flagged
Structural ignorance of gambling problems	Gambling Normalization	Everybody gambled, Gambling between deployments, Normalization of gambling
	Lack of Institutional Awareness: “You Have to Talk About It and Be Aware of It In Order to Do Anything”	Lack of awareness among veterans and military personnel to gambling as a potentially addictive behavior, Lack of awareness of GD in military and VA system, Supervisors awareness insufficient, The military did not raise awareness to gambling problems, Not enough assessment and screening for gambling, Lack of awareness during out processing, Asking the right questions about gambling is required
	“We’re Sorry, We Can’t Do Anything for You Here”	Did not know about treatment options, Missing clear directions to locate treatment options, It’s a matter of getting transportation, Bureaucracy, Strict inclusion criteria for GD treatment, Treating other mental health problems is more important, Treatment for other mental health conditions not GD, Private therapists are better, Easier talking to veteran therapists, Engaging in treatment, Not enough treatment options, Not enough remote therapy

### Trustworthiness of the Analysis

By adhering to Elo et al. (2014) criteria for trustworthiness, the following steps were conducted: Credibility was achieved through thorough familiarization with the data, involving repeated readings of the interview transcripts, with field notes added to the transcriptions after the interviews. Team collaboration played a significant role in enhancing the dependability and confirmability of the findings. Regular team meetings facilitated reflection on the codes, categories, and themes, ensuring consistency and reliability. MAXQDA software was used to manage and organize the data efficiently, facilitating the systematic handling of codes, memos, categories, and themes, thereby supporting the transferability and transparency of the results. Finally, to ensure that the findings consistently capture participants’ viewpoints, the authors present an abundance of participant quotations.

## Findings

The findings revealed two main themes reflecting that U.S. military culture and behavioral codes created barriers that hindered seeking treatment for GD. The first theme, *Emotional Suppression in Military Culture*, refers to deeply rooted cultural norms of military socialization that were internalized by the participants and conceptualized by the current study as internal barriers. The participants highlighted how the military’s emphasis on stringent emotional control and self-discipline created significant internal obstacles and expressed fear of repercussions for deviating from military expectations. The second theme, *Structural Ignorance of Gambling Problems,* highlighted external barriers unique to the U.S. military context. The interviewees described gambling as a normal activity among SMs, pointing to a systematic lack of awareness of gambling’s negative repercussions, reflected in inadequate training, insufficient screening, and a lack of specialized care for gambling issues.

Altogether, these themes delineated barriers before seeking help for GD and barriers while engaging in treatment. Most barriers were similar among veterans and SMs, and some were different between these groups. Veterans discussed their barriers as veterans but also recounted the barriers they faced as SMs.

### Emotional Suppression in Military Culture

The interviewees described their internal barriers to seeking help as deeply rooted in their military socialization. They emphasized that they were expected to show emotional control, restrain their vulnerabilities and maintain discipline in the military context of high-stress environments. Specifically, they believed that they had to be strong and having a gambling problem was a weakness. They also recounted their fear of potential repercussions if their GD was discovered.

#### “You Live Like a Strong and Not Weak”

The interviewees depicted their belief that they should remain strong and not show weakness, reflecting their internalization of a military culture that expects them to maintain a facade of strength and resilience. This pressure to uphold an appearance of control, discipline, and competence, even while struggling internally, amplified their reluctance to seek treatment and created feelings of shame, and embarrassment about their gambling issues. Cody (29, Black, male veteran, non-recovered) explained that his military training emphasized discipline and self-reliance, leading him to believe in his own strength. This belief made him reluctant to seek treatment for his GD:

Being in the military isn’t just about fighting, ‘Oh, I’m going to war.’ It builds your character. It makes you like more disciplined. You live like a strong and not weak so it felt like gambling was too less of a problem for me like I was bigger than that so I used to believe that I can like get rid of that problem by myself without asking for help.

Jared (33, Latino and Asian, male veteran, non-recovered) highlighted the military’s discouragement of seeking help for mental health problems:

In the military, we were taught to carry our own shit. The lone wolf attitude where I can do it myself. I don’t need anyone else. Let me just put the shit on me, and I’ll bear within, and I’ll finish it […]. The hardest thing for us is asking for help. You could not express yourself. […]. In the military they punished you if you seek help. Especially when it comes to mental health, like mental health. Oh, no! Hell to the no! They will punish you, and they’ll get you to the point where you’re like ‘You know what, it’s better not to even go to fucking therapy.’

#### “You Can Get It Flagged”

The interviewees also expressed fear that even if they were ready to discuss their gambling problems, doing so might lead to their issues being flagged. They were concerned that exposing a mental health problem could prevent them from performing their jobs, reduce their security clearance, and ultimately threaten their careers. Freya (31, White, female SM, non-recovered) revealed her fear of being flagged and facing career consequences as a barrier to seeking help:

I felt I didn’t want to share it with people from the military because first of all, you can get it flagged or I don’t know what even kind of consequences can be and I didn’t really want to play with this […] Confidential counseling session maybe, with mental health providers so soldiers wouldn’t feel afraid to actually go and discuss, what they’re doing, how they’re doing, and why they are doing and not being worried about how it’s going to affect their military careers. […] You sometimes feel like you’re just a number and you really mean nothing.

Similarly, Zach (27, White, male SM, recovered) shared his perception that the military’s approach to addressing mental health and addiction issues often leads to severe consequences that deter SMs from seeking help:

My biggest reason that I have not sought any kind of help is, given my background of military and law enforcement […] their first step is to it’s called DNA, and you get put on a ‘Do Not Arm list.’ So essentially you can’t carry weapons and therefore, you can’t do your job […] I think it’s a very bad process and it needs to be fixed. In my eyes, if I show any kind of fact that I had you know the gambling addiction or had the depression issues in the past, their immediate first reaction is to take away the one thing that you have is your job. I didn’t want to lose being able to do my job on a daily basis. So, I just kind of kept my mouth shut […] The confidential route would have been helpful cause then I wouldn’t have felt like I’d put my job at jeopardy as much […] Admitting any kind of at-risk behavior makes us kind of at peril to you know losing our job.

And Claire (56, African American, female veteran, non-recovered) concluded by calling for a much-required change in policy:

If you say something you’re kicked out, that’s it. You’re done. You have no more career. And I get it because the military is specifically designed for war. So, you don’t want some nut job with a weapon. But you still have to have an avenue for people to come forward and say, ‘Hey. I am not doing well. I’m not in a safe space.’

### Structural Ignorance of Gambling Problems

The interviewees recounted significant external barriers to treatment given the normalization and social legitimacy of gambling behaviors in military camps and the lack of awareness about the potential addictive elements of gambling. They argued that these barriers stemmed from a military culture that normalizes gambling, coupled with a structural deficiency to raise awareness of gambling as a potentially addictive behavior with harmful consequences. Therefore, when there is no awareness of a problem, there is no recognition of the need to offer help or encourage individuals to seek treatment. Moreover, the participants compared the awareness in the military system to alcohol and other drugs to the deficient awareness regarding gambling and criticized the VA for structural lack of awareness of their specific needs in the VA system as individuals with gambling problems, which lead to inadequate GD treatment.

#### Gambling Normalization

The veterans and SMs emphasized that gambling was a routine part of military life, considered a normal activity with widespread participation, especially in environments with limited recreational options, stationed near casinos, or with slot machines readily available on base. This diminished awareness of the potential harmful effects of gambling and the necessity of seeking help when gambling problems develop. Doug (59, White, male veteran, non-recovered) recounted how he was not aware of his gambling problem since this behavior was part of military life as a daily routine:

I had a gambling problem throughout my military. I just didn’t know it. I mean, we, I gambled. When I was stationed in XX back in the early eighties, we would get off work and we would start– first thing we would do, we would play like dice, then we play poker until the hours in the morning and I mean it was every day […] When in the war zone, I was on the mortuary teams, so I had to process remains and stuff to come back to the states. That’s horrific. After a while you’re just like a rubber ball. Your band’s stretched so tight and it’s going to snap. But we would get off, you know, have a few hours, we could have down time, and we would start playing poker […] You’re the new guy on the base and somebody’s inviting you over to play poker. So of course you’re gonna go, hell yeah I like to play poker. And it was that kind of thing you just kind of fed into it.

When Elliot (28, Black SM, Male, non-recovered), who also described gambling as part of military culture, was directly asked about barriers to treatment, he emphasized the influence of his army friends who gambled with him but did not see it as an issue.

[…] The fact that my friends have not seen or detected any issue with it. So it will feel weird that I’m the only member of the group, that out of five people, I’m the only one feeling that this is a problem that should be solved like this is an algebra that should be solved. So, yeah, I think those are kind of the major barriers.

#### Lack of Institutional Awareness: “You Have to Talk About It and Be Aware of It In Order to Do Anything”

The interviewees revealed a troubling oversight of the military and VA in failing to adequately warn veterans and SMs about the risks associated with gambling, suggesting a possible systemic deficiency in acknowledging and addressing gambling-related issues within these organizations, leaving veterans and SMs vulnerable to developing GD without sufficient preventive measures in place. For example, Madison (42, White, female veteran, non-recovered) noted the lack of discussion and awareness about gambling during her service, and advocated for increased awareness:

When I was in the military there was really no discussion about gambling. I think that there would probably be a good idea to bring more awareness to it. When I was in Europe, the military would scare the hell out of you to go to Red Light District cause of drugs, and people on the streets. But there was no discussion or awareness surrounding gambling.

Similarly, Sherry (39, Black, female veteran, non-recovered) criticized the lack of specific briefings on GD during her service:

[…] I had never really heard of it [gambling disorder]] [….] You do a buffillion briefings in the military. They do sexual assault. They do equal opportunity. They do alcohol use. They do a lot, but I’ve never had a briefing on gambling specifically

Britney (37, Black, female SM, non-recovered) said that there is screening for GD, but they (U.S. Armed Forces) treat all addictions the same:

They do ask you like typically, Do you have gambling problems? Do you have an alcohol problem? In the same way they ask, do you wear your seat belt when you drive? […] I don’t think it’s enough because of the military they treat all addictions the same, so for any addictive problem like just reading it off of some sheet because you know it’s a part of the intake questions. It isn’t enough because people feel like it’s trite, unoriginal, they don’t have any reason to answer authentically […] Getting a DUI [legal consequence of being caught driving under the influence of alcohol or drugs] and spending your whole 401K [retirement savings plan] are completely different things and shouldn’t be just looked at the same […] It’s just basically like there’s only one way for care and it’s like this cheesy checklist.

#### “We’re Sorry, We Can’t Do Anything for You Here”—no specific treatment for gambling problems

The interviewees claimed that many providers were unaware of available GD treatment resources. Treatment offered by the VA was generally not accessible, and when veterans could access treatment, they were mixed in with individuals struggling with SUDs, as opposed to receiving tailored solutions for GD.

Nate (59, White, male veteran, non-recovered) recounted his struggles in finding treatment tailored for GD in the VA system:

I didn’t know about treatment for vets and all that kind of stuff […] I called the GA hotline to see if I can get some help. There’s a guy […] he sent me the info and that’s what caused me to go to the Cleveland VA. Otherwise, I would have never known that they had a gambling treatment program for veterans […] You have to go and seek that help. It’s not just like, hey, here it is guys. Come and get it. No. It’s, you gotta find it. You definitely have to look and search for it. There are VAs that don’t even know it’s available in Cleveland. And there’s doctors within the VA that are like, (mocking VA voice) ‘Oh well, you know. Hey, just, you just got to control it. Just don’t worry about it. Go to a GA meeting. You’ll be fine. ‘ What are you telling somebody that for? You know, they really a lot of people just don’t get it. I think that’s the biggest thing.

Similarly, Grace’s (53, Multiracial, female veteran, non-recovered), who pointed out the lack of specific treatment to gambling, also mentioned the VA’s shortcomings by illustrating the challenges of maintaining consistent therapy due to high staff turnover and the inadequate treatment for GD:

The VA also has a high rate of turnover. So, I had to start over several times […] They sent me to rehab for trying to commit suicide, and they’re like, well, we’re sorry we can’t do anything for you here [in terms of GD], but we hope you enjoy your time here. I’m like that is not helpful […] People were talking about drugs. People were talking about alcohol. And I think that’s where I’m having a hard time. I feel like, those are like specific things, you know. So, gambling is a specific thing, that can be worked on. But I’m like, that’s not my problem, you know. I don’t have a problem with alcohol.

## Discussion

This study uncovered significant internal and external challenges to seeking help for GD within both the U.S. military and VA contexts, illustrating how environmental factors and individual experiences are intertwined and shape access to GD treatment (Hutchison, 2018). In the current study, internal barriers to accessing GD treatment were rooted in the military culture, largely stemming from the internalization of the socio-cultural context by veterans and SMs through their socialization to the military culture. Our first theme, *Emotional Suppression in Military Culture,* pertains to internalization process of military culture and largely corresponds with the influential work on emotional regulation written by Arlie Hochschild (1983), who argued that “Feeling Rules” are deeply embedded social and cultural guidelines. This internalization functions as a form of “deep etiquette,” guiding emotional responses and making them appear natural despite being socially constructed. For instance, while interviewees in the present study were able to describe their perceptions of GD as a weakness, they often struggled to recognize that this view is socially embedded within them. This finding aligns with other research demonstrating that mental health problems among military personnel are consistently described as a weakness preventing help seeking (Sharp et al., 2015; Silvestrini & Chen, 2023).

As for external barriers, the normalization of gambling, lack of structural awareness of gambling as addictive, inadequate screening methods to identify individuals with GD, and the lack of tailored treatment options for GD in the VA healthcare system were all identified as notable barriers to accessing GD treatment. Weber (1978) foundational analysis of organizations explains why military institutions necessitate higher degrees of discipline, order, and rational thought to fulfill their role in national defense and the organization of violence on behalf of the state. This observation distinguishes them from other institutions, such as law enforcement, which primarily regulates civilian affairs at the local level (Nuciari, 2018); this also extends to healthcare systems, which emphasize patient care and public health, requiring adaptability and responsiveness to diverse medical needs (Soeters, 2018). Ample studies have documented how strongly military culture stigmatizes mental health struggles, as they challenge the fundamental bureaucratic principles of rationality, objective decision-making, control, and efficiency (e.g., Mansoor & Murray, 2019; Reed, 2015). Moreover, Weber’s organizational analysis has formed a foundation for understanding how institutional culture constructs norms, and acts as a mechanism of external control shaping individual actions (Kunda, 1995).

Furthermore, Weick and Sutcliffe’s (2011) work employs the concepts of “mindlessness” and “normalization” to explain how both individual and structural levels of awareness can be compromised in organizations. They argued that when individuals or groups in institutional settings rely on established routines and past experiences, these routines and normalized activities become “mindless,” leading them to overlook or fail to question underlying assumptions. On a structural level, “normalization” involves the organizational tendency to categorize events and activities into familiar patterns, often overlooking unique or unusual elements, thus obscuring the recognition of emerging threats that do not fit established expectations, leading to inadequate structural responses. Hence, it is unsurprising that many SMs mindlessly gambled without recognizing its associated risks. At the structural level, the military’s bureaucratic tendency to operate according to known patterns probably inhibited it from recognizing risks associated with gambling and adapting to them. Such gaps in organizational knowledge may create profound implications for individuals and organizations, for example, the widespread gambling practices without the awareness of their harmful consequences among SMs and the organizational failure to provide adequate support and treatment for those affected by GD.

### Policy Recommendations

We propose several policy recommendations aimed at addressing these barriers, such as regular and comprehensive training sessions on the harmful consequences of gambling for veterans and SMs. With the passage of the National Defense Authorization Act for Fiscal Year 2019 (NDAA), the U.S. Armed Forces was required to screen SMs for GD in both the Annual Periodic Health Assessment and the Health Related Behavior Survey (H.R. 5515; 2018). The Department of Defense (DoD) issued guidance for healthcare providers to administer “the 3-question Brief Biosocial Gambling Screen” (included in the Periodic Health Assessment) to screen SMs for GD (DHA-PI 6200.07). While these policies are helpful, we propose that incorporating ongoing screening throughout many settings in DoD beyond a single annual evaluation would enable healthcare professionals to identify GD early and assist with referral services.

Notably, a recent meta-review concluded that given the reported low prevalence of GD and subclinical gambling problems among the SM population, routine screening may be unfeasible (Segura et al., 2024). The authors argued that screening efforts should instead be focused on higher-prevalence subpopulations (SMs already identified with substance use or mental health problems), concluding that routine screening of all SMs for GD is unwarranted. Conversely, other authors have suggested that the DoD should proactively screen all SMs for GD to protect the operational readiness, financial stability, mental health, and overall security of its personnel (Smith et al., 2024). We expect the debate around screening SMs for GD will continue, however, the current study uniquely speaks to the specific internal and external barriers that exist for SMs and veterans with GD, shedding some insights on where potential policy changes could be made within the U.S. Armed Forces.

For those who have developed GD, tailored treatment programs should be designed to meet their specific needs, considering unique circumstances such as deployment overseas and situations in which SMs may feel ashamed or reluctant to seek counseling. The policy must ensure a safe environment for disclosing gambling issues among SMs that also minimize career repercussions. Furthermore, we acknowledge that there are legitimate concerns around increased vulnerabilities and risks (i.e., national security) for the U.S. Armed Forces when SMs experience significant financial hardships due to GD. Therefore, the development of policies that foster early disclosure and help seeking among SMs are strongly recommended. The U.S. military should also actively encourage alternative leisure activities besides gambling, ensuring a range of accessible options for SMs while stationed outside of the U.S. Lastly, gambling facilities (e.g., legal slot machine parlors operated by the DoD on overseas military bases) need to be thoroughly evaluated to determine the relative risk they pose over the benefits they hold as entertainment for SMs.

### Limitations and Future Research

This study has several limitations. First, the sample included only four SMs (_age_ range-27–37) who were considerably younger than the veteran sample (_age_ range = 29–72). We found that the lived experiences of SMs and veterans varied widely, and the large age difference between them also reflected different eras of military service. Moreover, the difference in age between SMs and veterans should also note that many of the SMs grew up with access to various forms of digital technology, reflecting a possible difference in their increased familiarity with using internet-based technologies to facilitate help seeking. Although the U.S. Armed Forces has made progress with routine screening for mental health disorders among SMs, current barriers (e.g., stigma, career concerns) still remain (Hom et al., 2017). The degree of comfort SMs or veterans would experience if utilizing an internet-based treatment specific to GD remains unexplored, though we posit that such a resource could potentially reduce barriers around seeking helping.

The remaining participants were veterans who shared their experiences as veterans and former SMs, limiting our ability to fully uncover structural barriers related to tailored treatment for GD within the military system. Furthermore, there were instances when the interviewees did not specify the roles of the individuals responsible for screening and treatment given to them or their qualifications, limiting our ability to speak deeply about current gaps in healthcare settings. Although we included eight women, further research examining barriers to care for female veterans and SMs is needed. Future research should thoroughly investigate the specific barriers SMs face and their unique needs for care to ensure consistent care between the VA and DoD. Lastly, the high degree to which SMs use electronic devices (e.g., smartphones, computers) for online entertainment should be explored, particularly when thinking about mindlessness and its role in facilitating gambling problems or other addictive behaviors.

## Conclusion

The present findings reveal that barriers for seeking treatment in veterans and SMs are deeply rooted in U.S. military culture. Interviewees discussed both internal and external barriers to accessing care for GD. Collectively, the present findings highlight the necessity for cultural and institutional recognition of gambling as a potentially addictive behavior and the implementation of policies that aid veterans and SMs in accessing treatment for GD.

## Data Availability

Data will be made available on reasonable request.
